# Metabolic Signature of Hepatic Fibrosis: From Individual Pathways to Systems Biology

**DOI:** 10.3390/cells8111423

**Published:** 2019-11-12

**Authors:** Ming-Ling Chang, Sien-Sing Yang

**Affiliations:** 1 Liver Research Center, Division of Hepatology, Department of Gastroenterology and Hepatology, Chang Gung Memorial Hospital, Taoyuan 33305, Taiwan; 2 Department of Medicine, College of Medicine, Chang Gung University, Taoyuan 33305, Taiwan; 3 Liver Center, Cathay General Hospital Medical Center, Taipei 10630, Taiwan

**Keywords:** hepatic fibrosis, HSC, aerobic glycolysis, Fischer’s ratio, TCA cycle, transcriptomics, proteomics, metabolomics

## Abstract

Hepatic fibrosis is a major cause of morbidity and mortality worldwide, as it ultimately leads to cirrhosis, which is estimated to affect up to 2% of the global population. Hepatic fibrosis is confirmed by liver biopsy, and the erroneous nature of this technique necessitates the search for noninvasive alternatives. However, current biomarker algorithms for hepatic fibrosis have many limitations. Given that the liver is the largest organ and a major metabolic hub in the body, probing the metabolic signature of hepatic fibrosis holds promise for the discovery of new markers and therapeutic targets. Regarding individual metabolic pathways, accumulating evidence shows that hepatic fibrosis leads to alterations in carbohydrate metabolism, as aerobic glycolysis is aggravated in activated hepatic stellate cells (HSCs) and the whole fibrotic liver; in amino acid metabolism, as Fischer’s ratio (branched-chain amino acids/aromatic amino acids) decreases in patients with hepatic fibrosis; and in lipid metabolism, as HSCs lose vitamin A-containing lipid droplets during transdifferentiation, and cirrhotic patients have decreased serum lipids. The current review also summarizes recent findings of metabolic alterations relevant to hepatic fibrosis based on systems biology approaches, including transcriptomics, proteomics, and metabolomics in vitro, in animal models and in humans.

## 1. Introduction

Hepatic fibrosis, characterized by excessive extracellular matrix (ECM) deposition and fibrous scar formation in the liver [1,2], is a major cause of morbidity and mortality worldwide, as it ultimately leads to cirrhosis and hepatocellular carcinoma (HCC) [3]; cirrhosis is estimated to affect up to 2% of the global population [4,5]. A wide spectrum of chronic liver injuries, including viral hepatitis (hepatitis B and C), cholestatic liver diseases, alcohol abuse, nonalcoholic fatty liver disease (NAFLD), and nonalcoholic steatohepatitis (NASH), may cause chronic hepatic inflammation and ultimately lead to hepatic fibrosis [1]. However, no effective therapy other than liver transplantation is currently available for hepatic fibrosis. Recent studies indicate that hepatic fibrosis is reversible when the causative agent is removed [3]. When the removal of the underlying etiology is impossible, early identification and therapeutics targeting hepatic fibrogenesis are crucial for preventing the associated negative consequences. Hepatic fibrosis is diagnosed and confirmed by liver biopsy, and the erroneous nature of this technique necessitates the search for noninvasive alternatives [6]. Current biomarker algorithms include indirect surrogate measures, such as aminotransaminases and platelet count, and direct measures of fibrinogenesis or fibrinolysis, including hyaluronic acid and tissue inhibitor of metalloproteinase-1. The limitations of these biomarker models include an indeterminate range and a limited predictive ability, and their utilization requires knowledge of patient comorbidities, which may produce false results in a small proportion of individuals [7]. Due to universal hepatitis B immunization and the successful treatment of hepatitis C with direct-acting antivirals [8,9], the prevalence rates of both hepatitis B and C are decreasing, and the importance of metabolic liver diseases such as NAFLD and NASH as risk factors for hepatic fibrosis is rapidly increasing. Moreover, many metabolic and immune response pathways are evolutionarily conserved and are highly integrated as a central homeostatic mechanism [10], and the liver is the largest organ and is a major metabolic hub of the body [11,12,13]; thus, probing hepatic fibrosis-associated metabolic alterations holds promise for the discovery of new markers and therapeutic targets for hepatic fibrosis. In particular, by taking advantage of systems biology, an integrative discipline that connects molecular components to physiological functions and organismal phenotypes through quantitative reasoning, computational models and high-throughput experimental technologies [14], the metabolic alterations due to hepatic fibrosis can be surveyed comprehensively. Specifically, genetic polymorphisms identified in genome-wide association studies and novel proteins identified by proteomic technology offer the possibility of the further refinement and individualization of biomarkers of hepatic fibrosis [7]. Moreover, metabolomics, the identification and quantification of all or specified metabolites in a living system under a specific condition or disease [12,13,15], might be a powerful platform for discovering novel biomarkers and biochemical pathways to improve the diagnosis, prognostication and treatment of hepatic fibrosis [16]. The current review thus summarizes recent findings of metabolic alterations relevant to hepatic fibrosis from the aspects of individual pathways and systems biology, including transcriptomics, proteomics, and metabolomics in vitro, in animal models and in humans.

## 2. Hepatic Stellate Cells: Crucial Cells for Hepatic Fibrosis

HSCs are the primary cells associated with hepatic fibrosis; they comprise approximately 1/3 of nonparenchymal cells and 15% of total resident cells in the normal human liver. There are three broad components of the life cycle of activated HSCs, including quiescence, activation, and perpetuation. Interestingly, activated HSCs can partially reverse this phenotype, termed ‘reversion’, and promote the resolution of hepatic fibrosis [17]. Retinoic acid is a metabolite of vitamin A (retinol) that mediates the functions of vitamin A, which is generally required for growth and development [18]. The storage of retinoic acid in cytoplasmic droplets is a unique characteristic of quiescent HSCs, and retinoic acid storage is gradually lost during transdifferentiation [19]. Thus, HSC activation is characterized by the loss of the adipogenic phenotype and an increase in ECM expression. Extracellular signals from resident and inflammatory cells, including macrophages [20], as well as from the ECM itself modulate the severity of fibrosis. Fibronectin, a matrix protein modifies transforming growth factor β (TGFβ) availability [21], and the TGFβ pathway itself affects cellular metabolism.

## 3. Metabolic Pathways Associated with Hepatic Fibrosis

### 3.1. Carbohydrate-Associated Pathways

**Figure 1:**
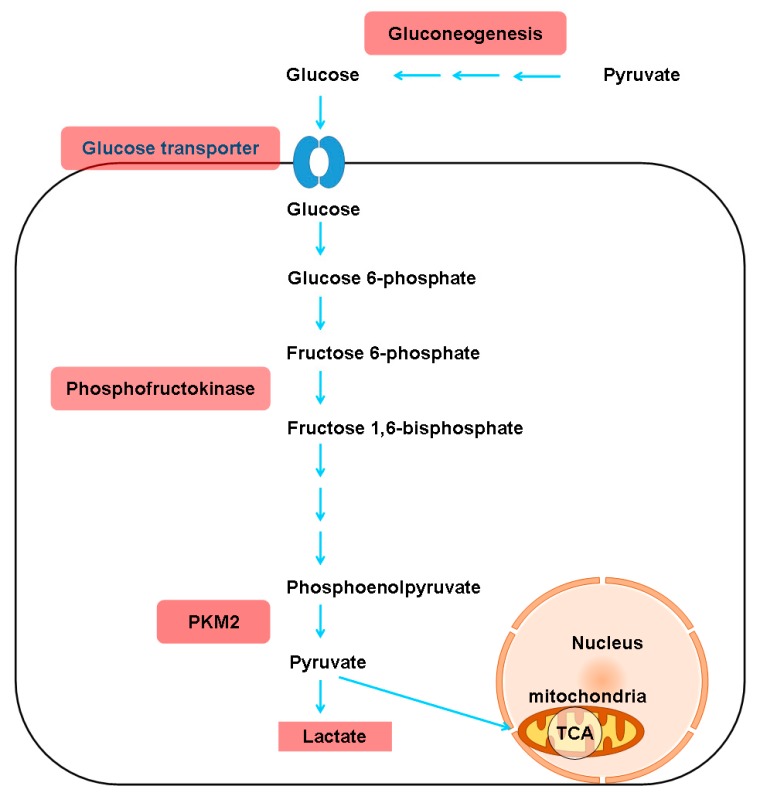
Alterations in carbohydrate metabolism associated with hepatic fibrosis in a representative liver cell. PKM2, pyruvate kinase M2; TCA, tricarboxylic acid cycle. Upregulated metabolites, enzymes, transporters or metabolites are shown in red boxes.

Emerging evidence suggests that HSCs utilize aerobic glycolysis during activation [22], and the inhibition of aerobic glycolysis blocks HSC contraction [23]. Moreover, the transdifferentiation of quiescent HSCs into myofibroblasts induces glycolysis and causes lactate (the end product of glycolysis) accumulation [24]. Consistently, both glucose transporter 1, which facilitates glucose transport across the plasma membranes of mammalian cells [25], and pyruvate kinase M2 (PKM2), which catalyzes the last step of glycolysis, are highly expressed in fibrotic liver samples and in exosomes derived from activated HSCs [26]. In addition, phosphofructokinase, a key regulator of glycolysis, catalyzes the phosphorylation of fructose-6-phosphate to generate fructose-1,6-bisphosphate, which is increased significantly in activated HSCs [27]. Exosomes released from HSCs are associated with the activation and glucose uptake of HSCs and affect the metabolic switch to glycolysis in liver nonparenchymal cells, including quiescent HSCs, Kupffer cells, and liver sinusoidal endothelial cells, via the delivery of glycolysis-related proteins [26]. As an extreme form of hepatic fibrosis, cirrhosis is a state of accelerated starvation with reduced hepatic glycogen availability and increased gluconeogenesis (an indicator of protein oxidation) [28] that requires amino acid diversion from other metabolic functions [29]. The levels of both lactate [30] and PKM2 [31] are increased in cirrhotic patients compared to controls and seem to increase with the severity of cirrhosis. Glucokinase (GCK) is the principal hexokinase (HK) in the liver, operating as a glucose sensor to regulate glucose metabolism and lipid homeostasis [32]. Decreased GCK activity might account for the decreased hepatic glycogen stores [33] in cirrhotic patients. Moreover, a study that surveyed 14 single nucleotide polymorphisms (SNPs) in adolescents with biopsy-proven NAFLD and in control individuals showed that the strongest variants associated with the severity of fibrosis were rs1260326 and rs780094 (both associated with glucokinase (hexokinase 4) regulator (GCKR)) as well as rs659366 (associated with mitochondrial uncoupling protein 2). The human patatin-like phospholipase domain-containing 3 (PNPLA3) SNP was associated with a portal pattern of steatosis, inflammation and fibrosis [34]. The alterations in carbohydrate metabolism associated with hepatic fibrosis are shown in Figure 1.

### 3.2. Amino Acid-Associated Pathways

**Figure 2:**
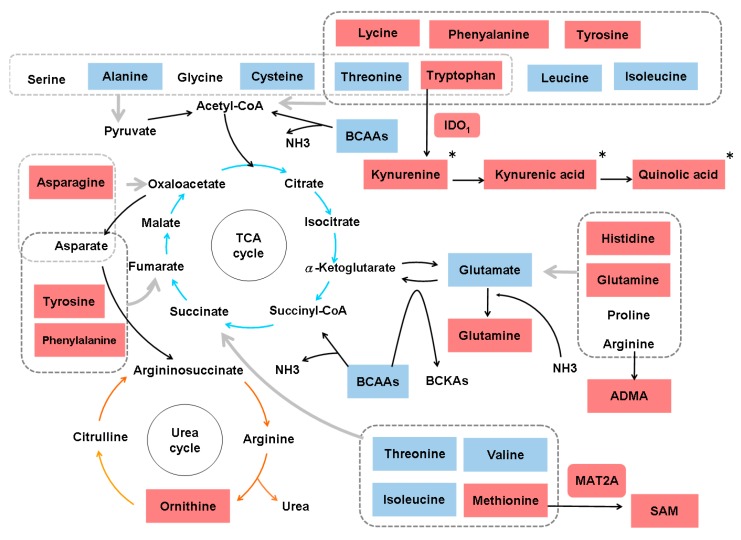
Alterations in amino acid metabolism associated with hepatic fibrosis. BCAAs, branched-chain amino acids; ADMA, asymmetric dimethylarginine; BCKAs, branched-chain alpha-keto acids; TCA, tricarboxylic acid cycle; MAT2A, methionine adenosyltransferase 2A; SAM, intracellular S-adenosylmethionine; IDO1, indoleamine 2,3-dioxygenase 1. *: activating features only noted in cirrhotic patients with acute decompensation and with acute-on-chronic liver failure. Gray arrows indicate the involvement of all amino acids in the indicated group. Upregulated metabolites are shown in red boxes, and downregulated metabolites are shown in blue boxes.

Studies in several animal models of hepatic fibrosis have revealed specific alterations in amino acid levels. For example, higher levels of plasma and hepatic asymmetric dimethylarginine (ADMA) were noted in bile duct-ligated rats than in control rats [35]. ADMA, an analog of L-arginine, is synthesized by protein methyltransferases, undergoes proteolytic degradation and competes with L-arginine for nitric oxide synthase, leading to malperfusion in various organs. Consistently, plasma ADMA levels are increased in cirrhotic patients [36]. In carbon tetrachloride (CCl_4_)-treated rats, increased levels of glutamine (GLN) and aromatic amino acids (AAAs) (phenylalanine and tyrosine), and decreased levels of branched-chain amino acids (BCAAs) (leucine, valine and isoleucine), glutamate (GLU), alanine and aspartate were found in plasma and muscles [37]. In another CCl_4_-induced cirrhotic rat model, increased levels of AAAs, aspartate, asparagine, methionine, ornithine and histidine and decreased levels of BCAAs, threonine and cystine were noted. An increase in most amino acids is characteristic of fulminant hepatic necrosis [38]. In yet another CCl_4_-treated rat model, two-dimensional (2D) differential in-gel electrophoresis (DIGE) analysis of liver proteins showed that 10 biomarkers, including GLN synthetase (GS), were associated with hepatic fibrosis [39]. In CCl_4_-treated mice, the TGFβ/p65/methionine adenosyltransferase 2A pathway was shown to regulate the intracellular S-adenosylmethionine concentration and hepatic fibrosis [40]. In thioacetamide (TAA)-treated rats, total essential amino acids and AAAs tended to increase, whereas BCAAs tended to decrease [41]. In another TAA-treated rat model, enzymes in primary metabolic pathways, such as BCAA metabolism, methionine breakdown and fatty acid β-oxidation, were downregulated. The upregulation of proteins related to oxidative stress and lipid peroxidation was also noted [42]. Generally, the amino acid molar ratio, called Fischer’s ratio (BCAAs/AAAs), is important for assessing liver metabolism, hepatic functional reserve and liver dysfunction severity [43], and a decreased Fischer’s ratio is characteristic of hepatic fibrosis or cirrhosis [38]. The enhanced consumption of BCAAs for ammonia detoxification to GLN in muscles leads to decreased BCAA levels in cirrhosis and urea cycle disorders [44]. Namely, GLN synthesis represents an alternative pathway for ammonia detoxification in cirrhotic patients, in contrast to the conversion of ammonia to urea in the liver via the tricarboxylic acid (TCA) cycle under physiological conditions [45]. However, whether GS is increased [46] or decreased [47] in cirrhotic patients remains unclear. BCAAs act as both protein substrates and key regulators of various nutrient metabolic processes; cirrhotic patients therefore suffer from various metabolic disorders after BCAA levels decrease [48]. On the other hand, AAAs and methionine are increased in cirrhotic patients due to portosystemic shunts and the reduced ability of diseased liver [49]. Consistently, an imbalance in plasma amino acids is observed in cirrhotic patients, and this imbalance suppresses the maturation of dendritic cells (DCs) by reducing intracellular ATP levels through interference with the mitochondrial TCA cycle [50]. Even in the early stages of NAFLD, hepatic and systemic hyperammonemia is evident due to reduced urea synthesis, and ammonia is known to activate HSCs and then accelerate hepatic fibrosis [51]. Moreover, argininosuccinic aciduria, the second most common urea cycle disorder, is caused by an argininosuccinate lyase deficiency and is associated with increased argininosuccinic acid levels, arginine deficiency and hepatic fibrosis [52]. Autoantibodies against aminoacylase-1 (ACY1) (an enzyme that participates in the urea cycle and the metabolism of amino groups) was identified as a marker of cirrhosis in patients with chronic hepatitis B (CHB) [53]. With regard to the kynurenine pathway, indoleamine 2,3-dioxygenase 1 (IDO1) is an intracellular rate-limiting enzyme involved in the metabolism of tryptophan, which subsequently mediates the immune response. A positive correlation between serum IDO1 levels and liver stiffness values was found in patients with CHB-related cirrhosis [54]. However, the levels of amino acids in the kynurenine pathway, including kynurenine, kynurenic acid and quinolinic acid, are increased in cirrhotic patients with acute decompensation, culminating in acute-on-chronic liver failure, but are normal in compensated cirrhotic patients [55]. The altered amino acid metabolism associated with hepatic fibrosis is outlined in Figure 2.

### 3.3. Lipid-Associated Pathways

The lipids present in hepatic HSC lipid droplets include retinyl ester, triglyceride, cholesteryl ester, cholesterol, phospholipids, and free fatty acids [56]. HSCs are the central cellular site for retinoid storage in healthy animals, accounting for as much as 50%–60% of total retinoids in the entire body [57]. Chronic liver diseases are often characterized by disturbed bile acid and vitamin A homeostasis; bile production is impaired, and HSCs lose vitamin A during transdifferentiation to myofibroblasts. Recent data revealed an intricate crosstalk between vitamin A metabolites and bile acids, in part through ligand-activated transcription factors and nuclear receptors, including retinoic acid receptor, retinoid X receptor and farnesoid X receptor; this crosstalk maintains vitamin A and bile acid homeostasis [58]. In addition, many peroxisome proliferator-activated receptor α (PPARα) target genes are involved in fatty acid metabolism in tissues with high oxidative rates, such as the muscle, heart and liver. PPARα activation, in combination with PPARβ/δ agonism, improves steatosis, inflammation and fibrosis [59]. The normal transcriptional function of PPARs contributes to the maintenance of HSCs in the quiescent phase. Reduced PPAR expression in HSCs strongly induces the progression of hepatic fibrosis and increases the production of collagen [60]. The levels of lipids, including total cholesterol, triglycerides, very low-density lipoprotein–cholesterol (VLDL), low-density lipoprotein–cholesterol (LDL), high-density lipoprotein–cholesterol (HDL) and total lipids, are lower in CHB cirrhotic patients than in control individuals, indicating hypolipidemia in cirrhotic patients [61], and the proteins downregulated in hepatitis B virus (HBV)-related hepatic fibrosis include apolipoproteins (apos) [62]. Consistently, the advanced fibrotic stage was associated with reduced apoA-I in HBV patients, and enrichment analysis highlighted the possible involvement of HDL-mediated lipid transport pathways [63]. HDL is an important endogenous inhibitor of inflammatory responses. The sera of cirrhotic patients showed reduced HDL levels and profoundly suppressed activities of several enzymes involved in HDL maturation and metabolism. HDL subclasses undergo a shift towards the larger HDL2 subclass. Moreover, apoA-I, apoC-III, apoE, paraoxonase 1 and acute phase serum amyloid A are altered in cirrhotic patients. Cholesterol efflux capacity appeared to be strongly associated with liver disease mortality [64]. The levels of lipid transfer inhibitor protein, apoJ and apoL-1 were decreased in patients with HCV-related cirrhosis [65]. However, apoA-IV levels were shown to increase in dimethylnitrosamine (DMN)-treated rats and in patients with hepatic fibrosis [66].

## 4. Metabolic Alterations Caused by Hepatic Fibrosis Based on Systems Biology Data

### 4.1. In Vitro Studies

Mass spectrometry-based stable isotope labeling by/with amino acids in cell culture (SILAC) analysis of an HSC cell line (LX-2) showed that the upregulated pathways associated with HSC reversion were mainly related to reducing oxidation and lipid [67], amino acid and glucose metabolism [68]. Lectin microarrays were used to probe the alterations in protein glycosylation in LX-2 cells, and the precise change in protein glycosylation was related to HSC activation [69]. A study on an in vitro HSC activation model that used high-throughput comparative proteomic analysis based on isobaric tags for relative and absolute quantitation (iTRAQ) labeling combined with online 2D nanoscale liquid chromatography and tandem mass spectrometry (2D nano-LC-MS/MS) showed that 200 downregulated proteins were primarily related to the immune response and lipid metabolism [70]. Another study that utilized microarray, quantitative polymerase chain reaction, and immunoblot analyses showed that the transdifferentiation of quiescent HSCs into myofibroblasts induced glycolysis and caused lactate accumulation. The increased expression of genes that regulate glycolysis required Hedgehog (Hh) signaling and involved the induction of HIF1α. Inhibitors of Hh signaling, HIF1α, glycolysis, or lactate accumulation promoted the conversion of myofibroblasts into quiescent HSCs [24].

### 4.2. Animal Studies

#### 4.2.1. CCl_4_-Treated Rats

An integrative analysis of transcriptomics and proteomics data based on rats treated with CCl_4_ for nine weeks identified alterations in 36 pathways, including retinol metabolism, PPAR signaling, glycolysis, gluconeogenesis, arachidonic acid metabolism, xenobiotic metabolism via cytochrome P450 and drug metabolism pathways. The expression of key targets such as cytochrome P450, family 4, subfamily a, polypeptide 3 (CYP3A4), aldehyde dehydrogenase 2, and aldehyde dehydrogenase 7 family member A1 (ALDH7A1) decreased after CCl_4_ treatment [71]. Microarray and Western blot analyses of CCl_4_-treated rats revealed the early induction of retinoic acid receptor responder protein 1 (RARRES1) mRNA and protein [72]. Ultra-performance liquid chromatography coupled to quadrupole time-of-flight mass spectrometry (LC-QTOF-MS) analysis of serum from CCl_4_-treated rats showed that β-muricholic acid and cervonoyl ethanolamide levels were significantly different in the fibrotic and control groups [73]. LC-QTOF-MS-based urinary and serum metabolic profiling of CCl_4_-treated rats revealed perturbations in tryptophan, valine, leucine, isoleucine, and TCA cycle metabolites, along with sphingolipid and glycerophospholipid metabolites, from the onset of hepatic fibrosis. The dysregulation of valine and bile acid biosynthesis metabolites occurred in the intermediate and advanced stages. Urinary kynurenic acid, 5-hydroxyindoleacetyl glycine, and 4-(2-amino-3-hydroxyphenyl)-2,4-dioxobutanoic acid and serum sphinganine, sphingomyelin (SM), l-leucine, l-tryptophan, and lysoPC(17:0) were changed at all time points [74]. Gas chromatography (GC)/MS analysis of liver metabolites in CCl_4_-treated rats showed alterations in pathways including glycolysis, gluconeogenesis, fructose and mannose metabolism, glycine, serine and threonine metabolism, lysine degradation, arginine and proline metabolism, glutathione metabolism, and sulfur metabolism [75]. GC-TOF-MS analysis of the urine metabolic spectrum in rats with early-stage hepatic fibrosis (CCl_4_ twice per week for four weeks) revealed potential biomarkers, including succinic acid, threonine, and lactose, that were associated with early-stage hepatic fibrosis [76]. ^1^H nuclear magnetic resonance (^1^H NMR) analysis of CCl_4_-treated rats identified seven urinary metabolites, including 2-oxoglutarate, citrate, dimethylamine, taurine, phenylacetylglycine, creatinine, and hippurate, as potential biomarkers of CCl_4_-induced chronic liver injury. The TCA cycle, gut microbiota metabolism, and taurine and hypotaurine metabolism were identified as the most affected metabolic pathways associated with chronic CCl_4_ hepatotoxicity [77]. GC/MS analysis of CCl_4_-treated rats identified altered serum metabolites, including isoleucine, L-malic acid, α-copper, carnitine, hippuric acid, glutaric acid and glucose, and altered urine metabolites, including 2-hydroxy butyric acid, isoleucine, *N*-acetyl-β-alanine, cytidine and corticoid. Hepatic fibrosis may be associated with the dysfunction of several metabolic pathways, including glucose, amino acid, P450, fatty acid, nucleic acid, electrolyte and glutathione biosynthesis [78].

#### 4.2.2. DMN-Treated Rats

An iTRAQ-based differential proteomic analysis performed in DMN-treated rats showed a decrease in key enzymes involved in fatty acid metabolism (e.g., acyl-CoA synthetase long chain family member 1), the TCA cycle (succinate dehydrogenase), glycogenolysis, and gluconeogenesis (pyruvate carboxylase and cytosolic phosphoenolpyruvate carboxykinase) and an increase in those involved in glycolysis (e.g., HK-1) [79].

#### 4.2.3. TAA-Treated Rats and Mice

An NMR-based metabolomic study investigating serum and urine samples from TAA-treated rats identified several potential serum biomarkers for hepatic fibrosis, including 2-hydroxybutyrate, 3-hydroxybutyrate and adipate in urine and phenylalanine, *N*,*N*-dimethyl glycine, *O*-acetyl glycoprotein, *N*-acetyl glycoprotein and choline in serum. Pathway analysis revealed disrupted signaling in the TCA cycle, pyruvate metabolism, starch and sucrose metabolism, glycolysis or gluconeogenesis, ketone body degradation, butanoate metabolism, and BCAA and AAA biosynthesis [80]. The analysis of serum samples from TAA-treated mice by LC and GC separation coupled with tandem mass spectrometry (MS-MS) showed that in addition to changes in serotonin and other vitamin A-related metabolites, the differences in metabolic products associated with hepatic fibrosis were related to energetics, redox homeostasis, bile acid production, inflammation, and other processes. For carbohydrate metabolism, a reduction in metabolic products associated with the TCA cycle was observed, suggesting the upregulation of glycolysis and a decrease in mitochondrial activity. Lipid metabolism showed an increase in ω-oxidation products, suggesting decreased β-oxidation [81].

#### 4.2.4. Rats Treated with Three Fibrogenic Compounds

The semiquantitative global MS analysis of plasma from male rats treated by oral gavage for five days with three fibrogenic compounds (allyl alcohol, CCl_4_, and 4,4’-methylenedianiline) showed that insulin-like growth factor-binding protein increased after the administration of a fibrogenic toxicant [82].

#### 4.2.5. Mouse Model of Primary Biliary Cholangitis

Comparative proteomics of purified mitochondria from a murine model of early PBC (constructed by the consecutive administration of poly(I:C)) using iTRAQ technology showed that most of the differentially expressed mitochondrial proteins between PBC mice and control mice were associated with reduced oxidation and lipid metabolism, and some were involved in the biosynthesis of steroid hormones and primary bile acids. Four proteins (hydroxyacyl-coenzyme A dehydrogenase, carnitine *O*-palmitoyltransferase 1, 2,4-dienoyl-CoA reductase, and isoform 1 of Enoyl-CoA hydratase domain-containing protein 2) involved in fatty acid β-oxidation were upregulated [83]. Ultra-performance liquid chromatography-linked electrospray ionization quadrupole time-of-flight MS (UPLC-ESI-QTOF-MS) and multivariate data analysis in a murine model of PBC generated with alpha-naphthylisothiocyanate showed that plasma bile acid and phospholipid levels increased while arginine and glutathione levels decreased [84].

A summary of the metabolic alterations associated with hepatic fibrosis based on animal systems biology is shown in Table 1.

### 4.3. Human Studies

#### 4.3.1. HBV-Related Hepatic Fibrosis

**Figure 3:**
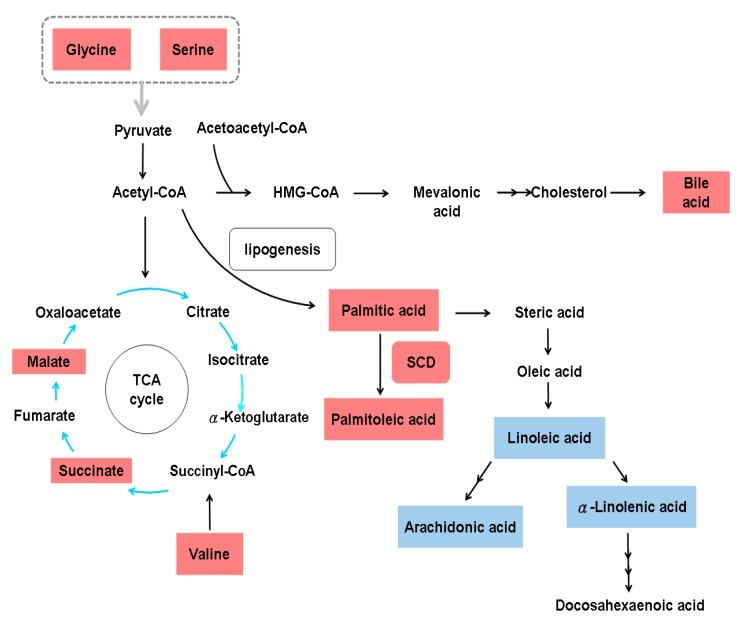
Metabolic alterations in hepatitis B virus-related hepatic fibrosis. HMG-CoA, 3-hydroxy-3-methyl-glutaryl-coenzyme A; SCD, stearoyl-coenzyme A desaturase; TCA, tricarboxylic acid cycle. The gray arrow indicates the involvement of all amino acids in the indicated group. Upregulated metabolites are shown in red boxes, and downregulated metabolites are shown in blue boxes.

Although activated HSCs undergo aerobic glycolysis [24], 2D electrophoresis and matrix-assisted laser desorption/ionization-TOF-MS (MALDI-TOF-MS) analysis of serum from eight patients with HBV infection (four with and four without hepatic fibrosis) showed decreased levels of enolase-1, a glycolytic enzyme, and increased levels of thrombospondin-1 in the samples from patients with hepatic fibrosis [85]. Through non-targeted metabolomics and targeted eicosanoid analysis of 139 serum samples from 49 HBV-related cirrhosis patients, 51 HCC patients and 39 healthy subjects, 42 metabolites were identified to be strongly associated with liver cirrhosis, including elevated fatty acids, glycine, serine, malic acid, succinic acid, valine and bile acids, and reduced carbohydrates, creatine, and uric acid [86]. The serum lipid profiles of 30 patients with HBV-related cirrhosis and 30 controls were generated by micro-lab and GC experiments, and these profiles showed a significant increase in palmitic and palmitoleic acid and a significant decrease in eicosatrienoic, arachidonic, linoleic and α-linolenic acids in cirrhotic patients. Indicators of stearoyl-CoA desaturase (SCD  =  ∆9-desaturase) activity, that is, the palmitoleic:palmitic acid (0.2) and oleic:stearic acid (1.5) ratios, were higher, while the PUFA:SFA ratio (0.6) was lower in HBV-related cirrhosis patients than in control subjects [61]. The metabolic alterations associated with HBV-related hepatic fibrosis based on systems biology data are shown in Figure 3.

#### 4.3.2. HCV-Related Hepatic Fibrosis

**Figure 4:**
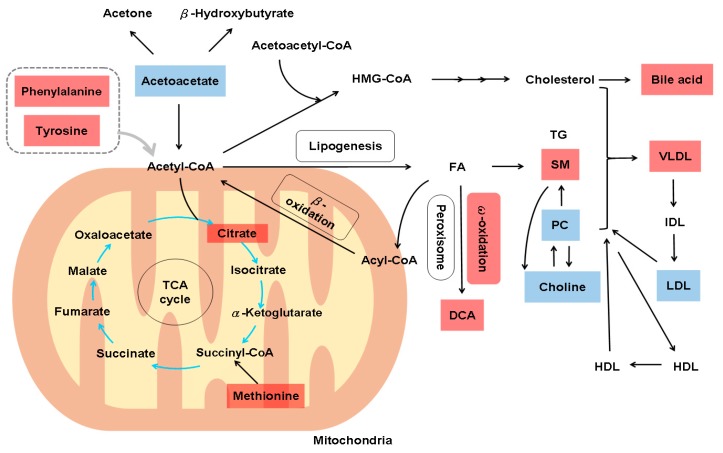
Metabolic alterations in hepatitis C virus-related hepatic fibrosis. In addition to decreasing Fisher’s ratio, hepatitis C virus-related hepatic fibrosis might lead to the impairment of mitochondrial processes, including impaired fatty acid oxidation, oxidative phosphorylation and responses to oxidative stress and reactive oxygen species. HMG-CoA, 3-hydroxy-3-methyl-glutaryl-coenzyme A; FA, fatty acids; TG, triglycerides; SM, sphingomyelin; PC, phosphatidylcholine; DCA, dicarboxylic acids; VLDL, very low-density lipoprotein-cholesterol; IDL, intermediate-density lipoprotein-cholesterol; LDL, low-density lipoprotein-cholesterol; HDL, high-density lipoprotein-cholesterol; TCA, tricarboxylic acid cycle; ROS, reactive oxygen species. Upregulated metabolites are shown in red boxes, and downregulated metabolites are shown in blue boxes.

A microarray study of 216 patients with HCV-related Child-Pugh A cirrhosis who were prospectively followed up for a median of 10 years showed that an adaptive metabolic shift from oxidative phosphorylation to glycolysis allowed for the maintenance of energy homeostasis during the early stages of liver injury but led to hepatocyte dysfunction during the terminal stages of chronic liver disease because hepatocytes could not sustain the high levels of energy production by glycolysis. This impairment corresponded to a decrease in the levels of the glucose-6-phosphatase catalytic subunit and phosphoglucomutase [87]. Consistently, an ultrasensitive proteomic study of liver tissue from 15 patients with HCV infection at different stages of fibrosis revealed the impairment of key mitochondrial processes, including fatty acid oxidation and oxidative phosphorylation, and responses to oxidative stress and reactive oxygen species in advanced-stage fibrosis (stage 3 to 4) [88]. The analysis of serum extracts from 203 patients with HCV infection (patients at METAVIR fibrosis stage F0–F1 (*n*  =  134) were categorized as “slow fibrosers”, and those at METAVIR F2–F4 (*n*  =  69) as “rapid fibrosers”) by reverse ultrahigh performance LC/MS showed that an algorithm consisting of two decreased SMs, SM(d18:2/16:0) and SM(38:1):SM(d18:1/20:0)+SM(16:1/22:0), and two increased phosphatidylcholines (PCs), PC(16:0/16:0) and PC(16:0/18:0), accurately classified rapid and slow fibrosers after liver transplantation. Moreover, specific bile acids and SMs increased notably with increasing hepatic fibrosis severity, thus differentiating between rapid and slow fibrosers, and Fischer’s ratio was decreased in rapid fibrosers [89]. Similarly, an integrated non-targeted metabolomics methodology employing both GC/MS and UPLC/MS-MS of the global serum metabolomes of 30 patients with HCC, 27 patients with HCV-associated cirrhosis and 30 healthy volunteers showed that increases in bile acids and dicarboxylic acids were highly correlated with cirrhosis [90]. Since dicarboxylic acids are found in identified intramitochondrial β-oxidation defects [12,91], aberrant dicarboxylic acid metabolism, enhanced bile acid metabolism and increased fibrinogen cleavage peptides may be signatures of cirrhosis [90]. ^1^H NMR spectroscopy of serum samples from HCV patients with (*n* = 27) or without hepatic fibrosis (*n* = 30) revealed lower levels of choline, acetoacetate and LDL in cirrhotic patients, and these three metabolites were the most informative biomarkers for predicting cirrhosis in HCV patients [92]. A study of plasma NMR spectra for two independent cohorts of chronic hepatitis C (CHC) patients and healthy controls (original study, *n* = 50; validation, *n* = 63) showed that increased fibrosis severity was associated with increased tyrosine, phenylalanine, methionine, citrate and VLDL and decreased creatine, LDL, PC, and *N*-acetyl-α1-acid-glycoprotein [93]. The metabolic alterations in HCV-related hepatic fibrosis based on systems biology data are outlined in Figure 4.

#### 4.3.3. NAFLD-Related Hepatic Fibrosis

Reverse-phase protein microarrays (RPMAs) of visceral adipose tissue collected from 167 NAFLD patients (training cohort, *n* = 117; test cohort, *n* = 50) showed that NASH was predicted by a model that included measurements of two components of the insulin signaling pathway: AKT kinase and insulin receptor substrate 1. The best-performing model for fibrosis relied on the levels of phosphorylated glycogen synthase kinase 3 (p-GSK-3) and of two subunits of cyclic AMP-regulated protein kinase A [94]. A high and ultrahigh-field magnetic resonance spectroscopy study of 30 NAFLD patients with nonalcoholic fatty liver (*n* = 8) or NASH (*n* = 22) revealed an increased phosphoethanolamine/total phosphorus (TP) ratio and a decreased glycerophosphocholine/(phosphomonoester+phosphodiester) ratio in advanced fibrosis. Moreover, the γ-ATP/TP ratio was decreased, while the phosphocreatine/TP ratio was increased in advanced fibrosis [95].

#### 4.3.4. Nonspecific Cirrhosis

**Figure 5:**
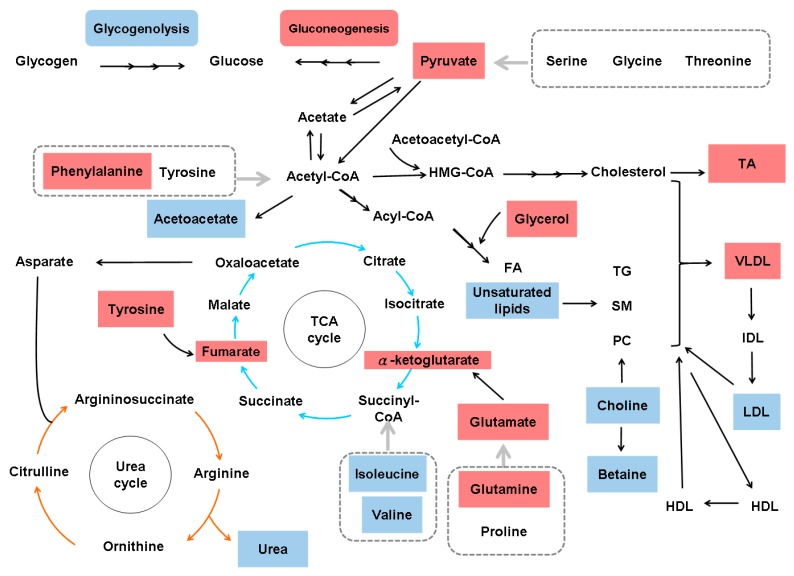
Metabolic alterations in nonspecific hepatic fibrosis. HMG-CoA, 3-hydroxy-3-methyl-glutaryl-coenzyme A; FA, fatty acids; TG, triglycerides; SM, sphingomyelin; PC, phosphatidylcholine; DCA, dicarboxylic acid; TA, taurocholic acid; VLDL, very low-density lipoprotein-cholesterol; IDL, intermediate-density lipoprotein-cholesterol; LDL, low-density lipoprotein-cholesterol; HDL, high-density lipoprotein-cholesterol; TCA, tricarboxylic acid cycle. Upregulated metabolites are shown in red boxes, and downregulated metabolites are shown in blue boxes.

An analysis of transcriptome data for the blood of 30 cirrhotic patients and eight healthy volunteers identified an active profibrotic transcriptomic program in cirrhotic patients involving ECM-receptor interactions, TGFβ signaling and cell adhesion molecules. This program coexists with alterations in the following pathways: glycine, serine and threonine metabolism, phenylalanine metabolism, tyrosine metabolism, ATP-binding cassette transport, purine metabolism and arachidonic acid metabolism [96]. The fecal metaproteomes of three cirrhotic patients with Child-Pugh scores of A, B, and C and of their spouses were surveyed by using a high-throughput approach based on denaturing polyacrylamide gel electrophoresis and LC/MS-MS, and the results showed that the proteins unique to cirrhosis were primarily involved in carbohydrate metabolism. Cirrhotic patients have unique BCAA, pantothenate, and CoA synthesis patterns, and these patterns were enhanced as the Child-Pugh score increased. The cirrhosis-related functional metabolites were mainly involved in carbohydrate, BCAA, pantothenate, and CoA metabolism, suggesting that the intestinal microbiota compensates for the fragile and nutrient-deficient body of cirrhotic patients [97]. An LC/MS-triple-quadrupole MS-based data acquisition mode (MRM) assay to quantify glycoforms of IgG subclasses 1–4 in five HCC patients, five cirrhotic patients, and five healthy individuals revealed an increase in galactose-deficient core fucosylated glycoforms in cirrhotic and HCC patients [98]. By using ^13^C and ^2^H_2_O NMR spectroscopy methods, net hepatic glycogenolysis and gluconeogenesis were examined in eight cirrhotic and four control subjects, revealing an increased gluconeogenesis rate and a decreased net hepatic glycogenolysis rate in cirrhotic patients compared with control subjects [99]. A cross-sectional, observational cohort study with an unbiased metabolomics analysis of 51 hospitalized cirrhotic patients (malnourished (42.3%) or nourished (57.7%)) showed that malnourished cirrhotic patients exhibited mild reductions in the skeletal muscle index and more marked reductions in the visceral fat index. The serum metabolite profiles showed reductions in multiple sphingolipid species in malnourished cirrhotic patients, suggesting that dysregulated sphingolipid metabolism might be involved in the pathophysiology of malnutrition in cirrhosis [100]. The GC/MS-based urine metabolomics profiles of 140 subjects, including 40 cirrhotic patients, 55 HCC patients and 45 healthy male subjects, showed differences in 8 metabolites, including glycine, serine, threonine, proline, citric acid, urea, xylitol, and arabinose, between the cirrhotic and the healthy groups [101]. High-performance liquid chromatography (HPLC) analysis of plasma samples from 388 chronic hepatitis or cirrhotic patients showed an imbalance in plasma amino acids in cirrhotic patients. Immature DCs showed reduced maturation and had lower intracellular ATP levels under conditions of advanced cirrhosis, despite the upregulation of mitochondrial respiratory chain complex genes; furthermore, TCA cycle metabolites, including fumarate and 2-oxoglutarate, were increased in DCs in the context of advanced cirrhosis [50]. LC/MS analysis of serum from 32 cirrhotic patients and 27 healthy volunteers showed that taurocholic acid was the most changed (increased) bile acid in cirrhotic patients [102]. Based on a raw metagenomic dataset and a metabolomic dataset of urine samples (generated using UPLC/MS) from 47 healthy controls and 49 compensated and 46 decompensated cirrhotic patients, the significantly reduced bacteria were found to be involved in the fermentation of plant cell wall polysaccharides, and resistant starch contributed to the reduced energy supply, the disorganized self-feeding and cross-feeding networks and the thriving of some opportunistic pathogens in the genus Veillonella. The marked decrease in butyrate-producing bacteria and the increase in *Ruminococcus gnavus* implicated in the degradation of mucus layer elements provide an explanation for impaired intestinal barrier function and systematic inflammation in cirrhotic patients [103]. Capillary electrophoresis and LC/MS metabolome profiling of serum samples from 248 patients with different liver diseases showed that γ-glutamyl dipeptides, which are biosynthesized through a reaction with γ-glutamylcysteine synthetase, indicated the production of reduced glutathione. The areas under the curve values of γ-glutamyl dipeptides in training and independent validation datasets were 0.803 and 0.993 in cirrhosis type C [104]. The metabolic profiles of HCC and cirrhotic patients generated by rapid resolution LC-QTOF-MS showed that glycocholic acid, glycochenodeoxycholic acid, taurocholic acid and taurochenodeoxycholic acid are potential biomarkers of cirrhosis [105]. One ^1^H NMR-based metabonomic study of 36 cirrhotic and 39 HCC patients showed that compared to the sera of healthy patients, the sera of cirrhotic and HCC patients had higher levels of acetate, *N*-acetylated glycoproteins, pyruvate, glutamine, alpha-ketoglutarate, glycerol, tyrosine, 1-methylhistidine and phenylalanine and lower levels of low-density lipoprotein, isoleucine, valine, acetoacetate, creatine, choline and unsaturated lipids [106]. Another ^1^H NMR-based metabonomic study of 18 stable cirrhotic patients, 18 patients with overt hepatic encephalopathy and 17 healthy volunteers showed that patients with cirrhosis had significantly impaired ketone body metabolism, urea synthesis and gluconeogenesis [107]. Moreover, a ^1^H-NMR metabolomic study of human liver specimens from liver donors (*n* = 16), patients with NASH (*n* = 14) and patients with alcohol-related liver damage (ARLD, *n* = 5) revealed that changes in BCAA homeostasis, the TCA cycle, purine biosynthesis intermediates and betaine were associated with the development of cirrhosis in both ARLD and NAFLD [108]. A review of metabolomic data showed that the liver develops a core metabolomic phenotype (CMP) that involves the dysregulation of bile acid and phospholipid homeostasis regardless of the provoking factor. The CMP commences at the transition between a healthy liver (phase 0) and NAFLD/NASH, alcoholic liver disease (ALD) or viral hepatitis (phase 1) and is maintained in the presence or absence of cirrhosis (phase 2) and HCC or cholangiocarcinoma development (phase 3). Both the Warburg shift from mitochondrial respiration to cytosolic glycolysis and the upregulation of fatty acid β-oxidation may occur as early as phase 1 [109]. The metabolic alterations in nonspecific hepatic fibrosis based on systems biology data are shown in Figure 5.

## 5. Concluding Remarks and Future Prospective

Mounting evidence shows that activated HSCs undergo aerobic glycolysis, and the whole fibrotic liver shifts from the TCA cycle to aerobic glycolysis for energy generation. The dominant enzymes and metabolites in glycolysis are consistently altered. The levels of many amino acids are increased in hepatic fibrosis, and Fisher’s ratios negatively correlate with the severity of hepatic fibrosis. Retinoic acid storage in cytoplasmic droplets within quiescent HSCs is gradually lost during HSC activation, and some serum lipid levels are decreased in patients with cirrhosis. Systems biology studies have shown that all carbohydrate-, amino acid- and lipid-associated pathways are altered in hepatic fibrosis and/or cirrhosis. Moreover, the dynamic evolution of systems biology has revealed that the progressive impairment of mitochondrial respiration during early cirrhosis and the subsequent decrease in energy production through glycolysis in failing cirrhotic livers as well as the Warburg shift to aerobic glycolysis that foreshadows HCC may occur as early as the initiation of NAFLD/NASH, ALD or viral hepatitis. Future work in prospective cohorts with verified hepatic fibrosis is needed to verify the metabolic signature and to identify therapeutic targets in hepatic fibrosis.

## Figures and Tables

**Table 1:** Summary of the metabolic alterations in hepatic fibrosis based on animal systems biology.

Fibrogenic Chemicals/Animals	Methods	Altered Pathways or Metabolites	Refs
CCl_4_/rats	Transcriptomics and proteomics	Pathways: retinol metabolism, PPAR signaling, glycolysis, gluconeogenesis, arachidonic acid metabolism, xenobiotic metabolism via cytochrome P450 and drug metabolism Enzymes: cytochrome P450, family 4, subfamily a, polypeptide 3; aldehyde dehydrogenase 2; and aldehyde dehydrogenase 7 family, member A1	[71]
	Microarray and Western blot	RARRES1 mRNA and protein	[72]
	LC-QTOF-MS	β-Muricholic acid and cervonoyl ethanolamide	[73]
	LC-QTOF-MS	Serum tryptophan, valine, leucine, isoleucine, TCA cycle metabolites, sphingolipid and glycerophospholipid metabolites, valine and bile acid metabolites Urinary kynurenic acid, 5-hydroxyindoleacetic acid, glycine, and 4-(2-amino-3-hydroxyphenyl)-2,4-dioxobutanoic acid and serum sphinganine, SM, l-leucine, l-tryptophan, and lysoPC(17:0)	[74]
	GC/MS	Pathways: glycolysis, gluconeogenesis, fructose and mannose metabolism, glycine, serine and threonine metabolism, lysine degradation, arginine and proline metabolism, glutathione metabolism, and sulfur metabolism	[75]
	GC-TOF-MS	Succinic acid, threonine and lactose	[76]
	^1^H NMR	Urinary 2-oxoglutarate, citrate, dimethylamine, taurine, phenylacetylglycine, creatinine and hippurate	[77]
	GC/MS	Serum metabolites: isoleucine, L-malic acid, α-copper, carnitine, hippuric acid, glutaric acid and glucose Urine metabolites: 2-hydroxy butyric acid, isoleucine, N-acetyl-β-alanine, cytidine and corticoid Pathways: glucose, amino acid, P450, fatty acid, nucleic acid, electrolyte and glutathione biosynthesis	[78]
DMN/rats	iTRAQ-based proteomic analysis	Key enzymes in fatty acid metabolism (acyl-CoA synthetase long chain family member 1), the TCA cycle (succinate dehydrogenase), glycogenolysis, and gluconeogenesis (pyruvate carboxylase and phosphoenolpyruvate carboxykinase in the cytosol) and an increase in glycolysis enzymes (HK-1)	[79]
TAA/rats	NMR-based metabolomics	Pathways: TCA cycle, pyruvate metabolism, starch and sucrose metabolism, glycolysis or gluconeogenesis, ketone body degradation, butanoate metabolism, and BCAAs and AAAs biosynthesis Serum metabolites: 2-hydroxybutyrate, 3-hydroxybutyrate and adipate in urine and phenylalanine, *N*,*N*-dimethyl glycine, *O*-acetyl glycoprotein, *N*-acetyl glycoprotein and choline in serum	[80]
TAA/mice	LC and GC separation coupled with MS-MS	Pathways: glycolysis and β-oxidation Metabolites: Serotonin and other vitamin A-related metabolites; metabolic products related to energetics, redox homeostasis, bile acid production, inflammation, and other processes	[81]
allyl alcohol, CCl_4_, and 4,4’-methylenedianiline/rats	MS	Insulin-like growth factor-binding protein	[82]
poly(I:C)/mice	iTRAQ	Mitochondrial proteins associated with reduced oxidation, lipid metabolism, and steroid hormone and primary bile acid biosynthesis. Four proteins (hydroxyacyl-coenzyme A dehydrogenase, carnitine O-palmitoyltransferase 1, 2,4-dienoyl-CoA reductase, and isoform 1 of Enoyl-CoA hydratase domain-containing protein 2) involved in fatty acid β-oxidation	[83]
alpha-naphthylisothiocyanate/mice	UPLC-ESI-QTOF-MS	Plasma bile acids, phospholipids, arginine and glutathione	[84]

Refs: references; CCl_4:_ carbon tetrachloride; PPAR: peroxisome proliferator-activated receptor; RARRES1: retinoic acid receptor responder protein 1; LC-QTOF-MS: Ultra-performance liquid chromatography coupled to quadrupole time-of-flight mass spectrometry; TCA: tricarboxylic acid cycle; GC/MS: gas chromatography–mass spectrometry; ^1^H NMR: ^1^H nuclear magnetic resonance; DMN: dimethylnitrosamine; iTRAQ: isobaric tags for relative and absolute quantitation; BCAAs: branched-chain amino acid; AAAs: aromatic amino acid; MS-MS: tandem mass spectrometry; UPLC-ESI-QTOF-MS: ultra-performance liquid chromatography-linked electrospray ionization quadrupole time-of-flight mass spectrometry.
